# Supplementary material for: Integrative analysis identifies gene signatures mediating the effect of DNA methylation on asthma severity and lung function
Source: Clin Epigenetics. 2024 Jan 20;16:15. doi: 10.1186/s13148-023-01611-9 (PMC10800055; doi:10.1186/s13148-023-01611-9)
Supplement: Supplementary file 1 — Additional file 1. Supplementary Figures S1–S6. [file 13148_2023_1611_MOESM1_ESM.docx]

**Integrative analysis identifies gene signatures mediating the effect of DNA methylation on asthma severity and lung function**

Eskezeia Y. Dessie^1^, Lili Ding^1^, Tesfaye B. Mersha^1^*

^1^Department of Pediatrics, Cincinnati Children’s Hospital Medical Center, University of Cincinnati College of Medicine, Cincinnati, OH, USA


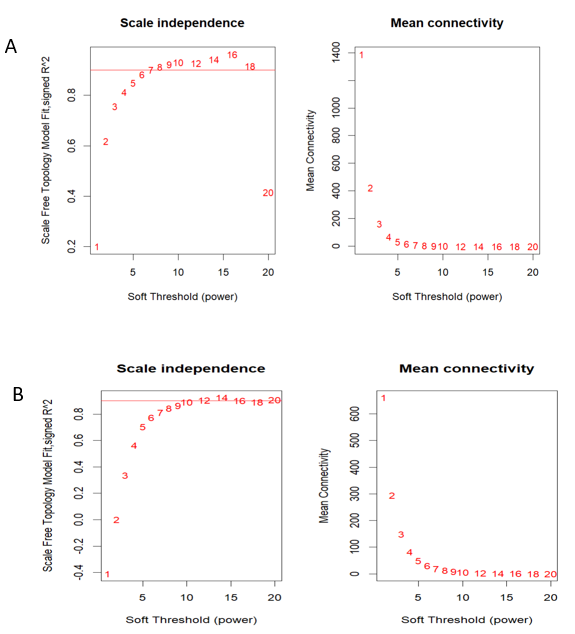


**Supplementary Figure S1:** WGCNA analyses to select soft thresholding power for a) DNA methylation derivation set b) RNA-seq derivation set. For DNA methylation data, co-methylation network analysis was constructed based on the suitable soft threshold power (β) = 7, which resulted scale free topology model fit signed topology R2 = 0.9. For RNA-seq data, co-expression network analysis was constructed based on the suitable soft threshold power (β) = 10, which resulted scale free topology model fit signed topology R2 = 0.9.


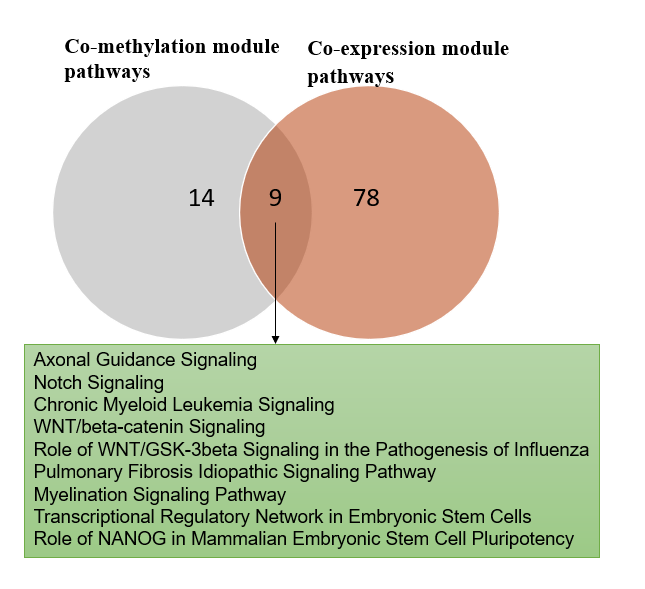


**Supplementary Figure S2:** Enriched shared pathways of genes in asthma-severity associated Co-methylation modules and genes co-expression modules.


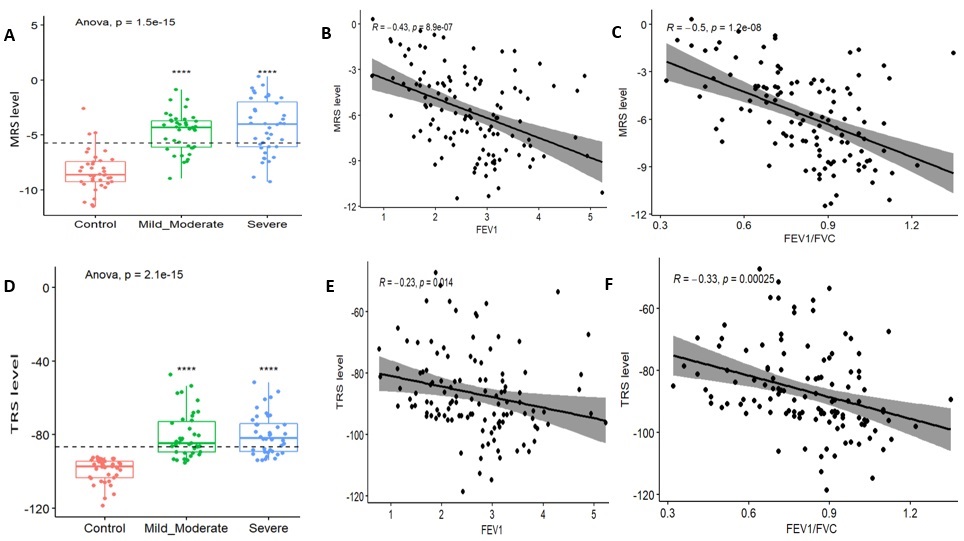


**Supplementary Figure S3:** Asthma severity and lung function measures (FEV1 and FEV1/FVC) association with risk score models in the derivation set. A) The risk score level plotting with asthma severity categories. B). The MRS plotted against FEV1. C). The MRS score plotted against FEV1/FVC. MRS- methylation based risk score. D) The TRS level plotting with asthma severity categories. E). The TRS plotted against FEV1. F). The TRS score plotted against FEV1/FVC. TRS-transcriptomic based risk score.


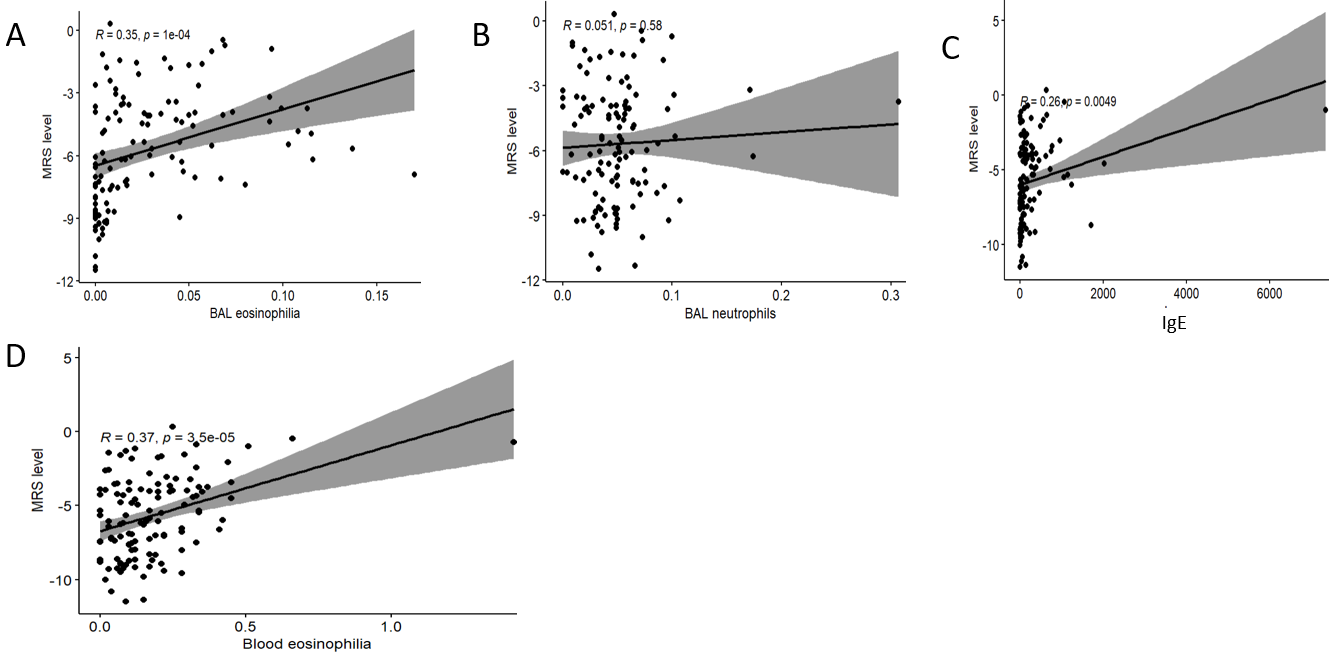


**Supplementary Figure S4**: The association between MRS level with various clinical measures including BAL eosinophilia, BAL neutrophils, IGE and blood eosinophilia in the methylation derivation set.


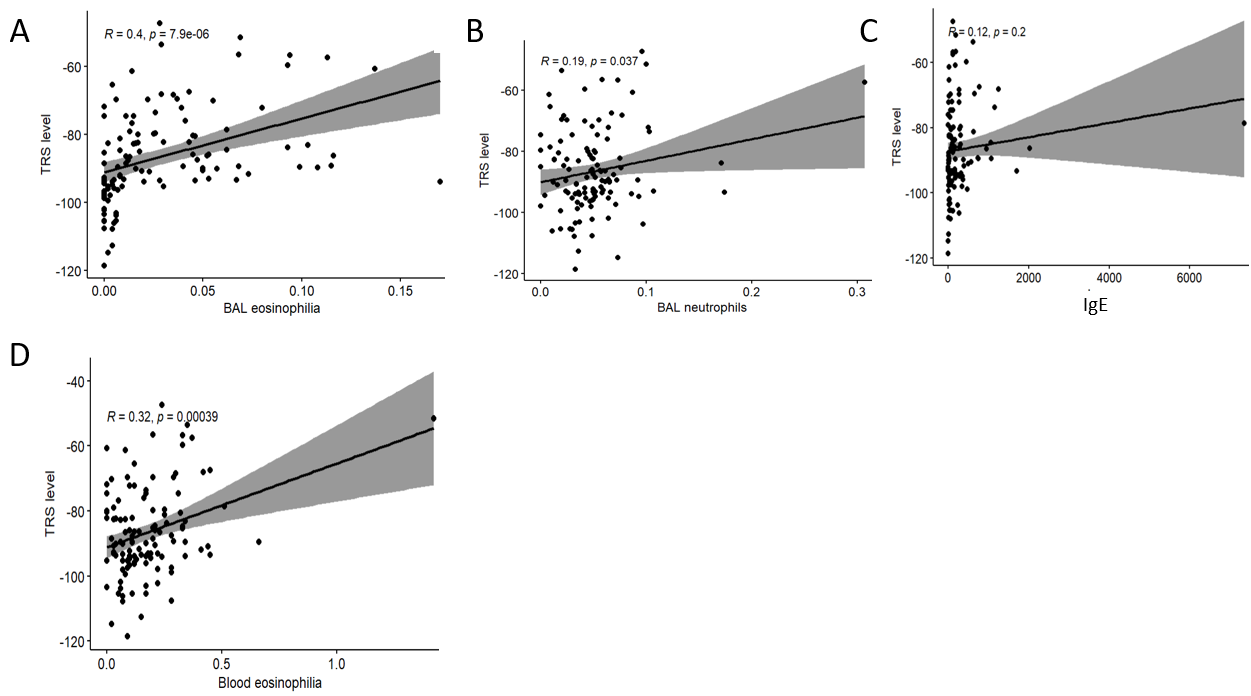


**Supplementary Figure S5**: The association between TRS level with various clinical measures including BAL eosinophilia, BAL neutrophils, IGE, and blood eosinophilia in the RNA-seq derivation set.


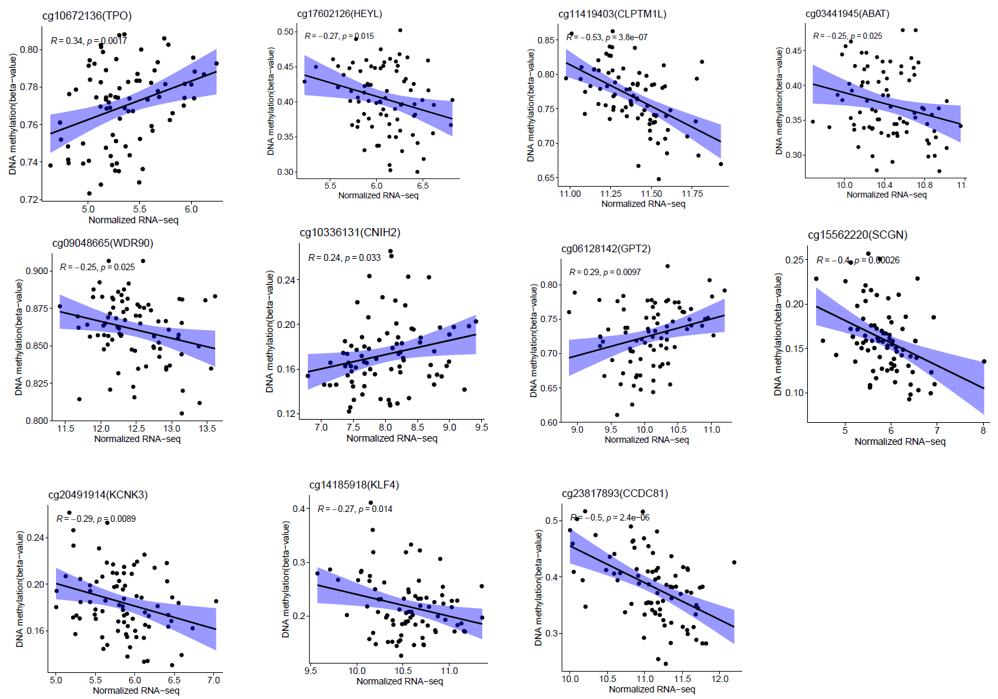


**Supplementary Figure 6:** Pairwise correlation between DNA methylation of CpG sites and expression annotated DEGs in AECs of validation set of asthmatic and control subjects.
