# Supplementary material for: Integrative analysis identifies gene signatures mediating the effect of DNA methylation on asthma severity and lung function
Source: Clin Epigenetics. 2024 Jan 20;16:15. doi: 10.1186/s13148-023-01611-9 (PMC10800055; doi:10.1186/s13148-023-01611-9)
Supplement: Supplementary file 2 — Additional file 2. Supplementary Tables S1–S10. [file 13148_2023_1611_MOESM2_ESM.docx]

**Integrative analysis identifies gene signatures mediating the effect of DNA methylation on asthma severity and lung function**

Eskezeia Y. Dessie^1^, Lili Ding^1^, Tesfaye B. Mersha^1^*

^1^Department of Pediatrics, Cincinnati Children’s Hospital Medical Center, University of Cincinnati College of Medicine, Cincinnati, OH, USA

**Supplementary Table S1**: Co-methylation module for asthma. A total of 1845 differentially methylated CpG sites (DMCs) with adjusted p-value <0.05 and absolute effect size (|∆_meth|) > 1% were clustered into four modules based on WGCNA. DMCs that were not correlated and assigned to a grey module were not considered for downstream analyses. The CpG sites in co-methylation modules that are associated with asthma severity and lung function are shown in Figure 2C. DEGs was defined based absolute value of log2(Fold change) > 0.1 and Benjamini and Hochberg based adjusted p-value < 0.05.

| **Co-methylation modules** | | | |
| --- | --- | --- | --- |
| **Module** | **Module size (CpGs)** | **N genes in the module** | **N genes with DEG padj <0.05** |
| Blue | 327 | 205 | 22 (11%) |
| Brown | 95 | 70 | 13(19%) |
| Turquoise | 1382 | 723 | 74(10%) |

**Supplementary Table S2**. List of significant pathways for genes that were correlated with the asthma-associated turquoise, brown and blue co-methylation module eigenvectors. The correlated methylated CpG sites mapped genes of each module with p-value < 0.01were included as input for pathway analysis.

| **Turquoise module (723 correlated methylated CpG sites mapped genes)** | | | |
| --- | --- | --- | --- |
| *Ingenuity Canonical Pathways* | P-value | Adjusted p-value | Molecules |
| RHO GTPase cycle | 3.24E-09 | 2.95E-06 | ABCD3,ABI2,ACBD5,ARHGAP28,ARHGAP29,  ARHGAP42,ARHGAP5,ARHGEF12,ARHGEF26,  ARL13B,BLTP3B,CCDC115,CDC42BPA,CFTR,CPD,  CPNE8,FAM169A,FILIP1,FRS2,GJA1,HSP90AA1,  HSP90AB1,KIDINS220,KTN1,LEMD3,MACO1,MPP7,  MTR,MYO9A,PAK2,PARD6B,PCDH7,PREX2,PTPN13,  RNF20,RRAS2,SH3RF1,TMPO,TOR1AIP1,VAPB,VAV3 |
| Generic Transcription Pathway | 1.2E-05 | 0.003631 | ESRRG,NR1D2,NR3C1,NR3C2,RBPJ,ZFP14,ZFP90,  ZIK1,ZKSCAN1,ZNF141,ZNF154,ZNF33B,ZNF382,  ZNF418,ZNF430,ZNF470,ZNF486,ZNF549,ZNF551,  ZNF568,ZNF610,ZNF619,ZNF627,ZNF660,ZNF667,  ZNF671,ZNF677,ZNF678,ZNF713,ZNF770,  ZNF772,ZNF773 |
| Transcriptional Regulatory Network in Embryonic Stem Cells | 2.82E-05 | 0.004571 | BMPR1A,BMPR1B,BMPR2,FZD4,FZD7,GSK3B,IL6ST,  LIFR,MAPK1,MEIS1,PIK3R4,RRAS2,SET,SMAD2,  SMAD5,TGFBR3,WNT5A |
| Class I peroxisomal membrane protein import | 3.02E-05 | 0.004571 | ABCD2,ABCD3,ACBD5,PEX12,PEX19,PEX3 |
| Human Embryonic Stem Cell Pluripotency | 3.72E-05 | 0.004571 | BMP3,BMPR1A,BMPR1B,BMPR2,FOXO1,FZD4,  FZD7,GSK3B,HSP90AA1,HSP90AB1,ID4,LIFR,  MAPK1,PIK3R4,PRKCE,RRAS2,SMAD2,  SMAD5,WNT5A |
| Mouse Embryonic Stem Cell Pluripotency | 3.98E-05 | 0.004571 | BMPR1A,BMPR2,FZD4,FZD7,GSK3B,HNF1A,  ID4,IL6ST,LIFR,MAPK1,PIK3R4,RRAS2,SMAD5 |
| Xenobiotic Metabolism Signaling | 4.07E-05 | 0.004571 | ALDH1A1,ALDH5A1,ALDH6A1,ALDH9A1,ARNT,  CAMK2D,ESD,FMO2,FMO3,GSTA1,GSTA2,GSTA3,  HS2ST1,HSP90AA1,HSP90AB1,MAP3K5,MAPK1,  NCOA1,PIK3R4,PPM1L,PPP2CB,  PPP2R5E,PRKCE,RRAS2 |
| WNT/beta-catenin Signaling | 5.5E-05 | 0.005012 | BMPR2,CDH12,CDH2,CSNK1G1,CSNK2A1,  CSNK2A2,FZD4,FZD7,GJA1,GSK3B,HNF1A,LRP6,  PPM1L,PPP2CB,PPP2R5E,TGFBR3,WNT5A |
| Mitotic Roles of Polo-Like Kinase | 5.75E-05 | 0.005012 | ANAPC1,CDC16,HSP90AA1,HSP90AB1,PPM1L,  PPP2CB,PPP2R5E,RAD21,SMC3,WEE1 |
| Role of NANOG in Mammalian Embryonic Stem Cell Pluripotency | 6.17E-05 | 0.005012 | BMP3,BMPR1A,BMPR1B,BMPR2,FZD4,FZD7,  GSK3B,IL6ST,LIFR,MAPK1,PIK3R4,RRAS2,  SMAD5,WNT5A |
| Xenobiotic Metabolism CAR Signaling Pathway | 6.61E-05 | 0.005012 | ALDH1A1,ALDH5A1,ALDH6A1,ALDH9A1,  FMO2,FMO3,GSTA1,GSTA2,GSTA3,HS2ST1,  HSP90AA1,HSP90AB1,MAPK1,NCOA1,PPM1L,  PPP2CB,PPP2R5E,PRKCE |
| Xenobiotic Metabolism AHR Signaling Pathway | 0.000117 | 0.007943 | ALDH1A1,ALDH5A1,ALDH6A1,ALDH9A1,  ARNT,GSTA1,GSTA2,GSTA3,HSP90AA1,  HSP90AB1,NCOA1 |
| PTEN Signaling | 0.000123 | 0.007943 | BMPR1A,BMPR1B,BMPR2,CSNK2A1,  CSNK2A2,FOXO1,FOXO3,GSK3B,INSR,ITGA2,MAPK1,  PREX2,PTK2,RRAS2,TGFBR3 |
| Role of Osteoblasts, Osteoclasts and Chondrocytes in Rheumatoid Arthritis | 0.0002 | 0.011749 | BMP3,BMPR1A,BMPR1B,BMPR2,CSNK1G1,  FOXO1,FZD4,FZD7,GSK3B,HNF1A,IL33,LRP6,  MAP3K5,MAPK1,NFATC3,PIK3R4,SMAD5,TAB2,WNT5A |
| Aryl Hydrocarbon Receptor Signaling | 0.000219 | 0.012303 | ALDH1A1,ALDH5A1,ALDH6A1,ALDH9A1,  ARNT,GSTA1,GSTA2,GSTA3,HSP90AA1,  HSP90AB1,MAPK1,NCOA7,NFIA,NFIB,RBL2 |
| PXR/RXR Activation | 0.00024 | 0.012589 | ALDH1A1,FOXA2,FOXO1,GSTA1,GSTA2,  INSR,NCOA1,NR3C1,PRKAR2B |
| Regulation of the Epithelial-Mesenchymal Transition Pathway | 0.000251 | 0.012589 | APH1B,CDH12,CDH2,FRS2,FZD4,FZD7,  GSK3B,HNF1A,MAPK1,MET,PARD6B,PIK3R4,  PYGO1,RBPJ,RRAS2,SMAD2,WNT5A |
| NFE2L2 regulating anti-oxidant/detoxification enzymes | 0.000282 | 0.01349 | GCLM,GSTA1,GSTA2,GSTA3,TXNRD1 |
| Cellular response to heat stress | 0.000309 | 0.013804 | CAMK2D,EEF1A1,GSK3B,HSP90AA1,  HSP90AB1,HSPA4L,MAPK1,  NUP133,NUP153,  RPA1,ST13 |
| Bile acid and bile salt metabolism | 0.000427 | 0.018197 | CH25H,CYP39A1,NCOA1,OSBPL6,  OSBPL9,SLC27A2 |
| STAT3 Pathway | 0.000468 | 0.018621 | BMPR1A,BMPR1B,BMPR2,IFNAR1,IL20RA,  IL6ST,INSR,MAP3K20,MAPK1,RRAS2,  SOCS5,SOCS6,TGFBR3 |
| HIPPO signaling | 0.000468 | 0.018621 | CRB1,PPM1L,PPP2CB,PPP2R5E,SAV1,SMAD2,S  MAD5,TEAD1,TP53BP2,YAP1 |
| Iron homeostasis signaling pathway | 0.000537 | 0.019953 | ABCB10,ARNT,BMP3,BMPR1A,BMPR1B,  BMPR2,CP,MAPK1,SLC11A2,SLC40A1,  SMAD2,SMAD5,TWSG1 |
| Chaperone Mediated Autophagy | 0.000589 | 0.02138 | ARL13B,CFTR,EEF1A1,HSP90AA1,HSP90AB1 |
| IGF-1 Signaling | 0.000661 | 0.022909 | CSNK2A1,CSNK2A2,FOXO1,FOXO3,MAPK1,  PIK3R4,PRKAR2B,PTK2,RRAS2,SOCS5,SOCS6 |
| Role of WNT/GSK-3beta Signaling in the Pathogenesis of Influenza | 0.000933 | 0.031623 | CSNK1G1,FZD4,FZD7,GSK3B,HNF1A,  IFNAR1,NCOA1,NCOA4,WNT5A |
| Formaldehyde Oxidation II (Glutathione-dependent) | 0.001047 | 0.033113 | ADH5,ESD |
| Role of BRCA1 in DNA Damage Response | 0.001122 | 0.033113 | ARID2,BRD7,E2F6,FANCF,HLTF,  MSH2,RBL2,RFC1,RPA1 |
| Pulmonary Fibrosis Idiopathic Signaling Pathway | 0.001148 | 0.033113 | AEBP2,BMPR2,CDH2,EFNB2,FOXO1,FOXO3,  FZD4,FZD7,GSK3B,HNF1A,IL6ST,ITGA2,MAPK1,  PIK3R4,PTK2,RBPJ,RPS6KA5,RRAS2,  SMAD2,TGFBR3,WNT5A,YAP1 |
| Myelination Signaling Pathway | 0.001148 | 0.033113 | APH1B,ARHGAP5,ASCL1,BMP3,BMPR1A,  BMPR1B,BMPR2,FZD4,FZD7,GSK3B,ID4,  ITGA2,MAPK1,MBP,NFATC3,PIK3R4,PRKAR2B,  PTK2,RRAS2,SMAD2,SMAD5,WNT5A |
| Amyloid Processing | 0.00123 | 0.034674 | APH1B,CSNK2A1,CSNK2A2,GSK3B,MAPK1,  PRKAR2B,PRKCE |
| Transport of bile salts and organic acids, metal ions and amine compounds | 0.001479 | 0.040738 | CP,SLC11A2,SLC13A2,SLC13A3,SLC22A4,  SLC39A10,SLC39A6,SLC40A1,SLC44A5 |
| Cardiomyocyte Differentiation via BMP Receptors | 0.001585 | 0.041687 | BMPR1A,BMPR1B,BMPR2,MEF2C,SMAD5 |
| Xenobiotic Metabolism PXR Signaling Pathway | 0.00182 | 0.046774 | ALDH1A1,ALDH5A1,ALDH6A1,ALDH9A1,  CAMK2D,ESD,GSTA1,GSTA2,GSTA3,HS2ST1,  HSP90AA1,HSP90AB1,NCOA1,PRKAR2B,PRKCE |
| FOXO-mediated transcription of cell cycle genes | 0.001862 | 0.046774 | FOXO1,FOXO3,RBL2,SMAD2 |
| Adipogenesis pathway | 0.00195 | 0.047863 | BMPR1A,BMPR1B,BMPR2,FOXO1,FZD4,  FZD7,KAT6B,KAT7,RUNX1T1,SENP2,  SMAD5,WNT5A |
| Sertoli Cell-Germ Cell Junction Signaling Pathway (Enhanced) | 0.002089 | 0.048978 | CDH2,CLDN16,CLDN8,GJA1,GSK3B,ITGA2,  MAP3K5,MAPK1,PIK3R4,PPM1L,  PPP2CB,PPP2R5E,  PRKAR2B,PTK2,RRAS2,SMAD2,SP3 |
| NRF2-mediated Oxidative Stress Response | 0.002188 | 0.048978 | ABCC4,CYP2U1,DNAJB4,DNAJC5B,GCLM,  GSK3B,GSTA1,GSTA2,GSTA3,HSP90AA1,  HSP90AB1,MAP3K5,MAPK1,PIK3R4,  PRKCE,RRAS2,TXNRD1 |
| EGR2 and SOX10-mediated initiation of Schwann cell myelination | 0.002188 | 0.048978 | ADGRV1,MBP,TEAD1,UTRN,YAP1 |
| Ovarian Cancer Signaling | 0.002239 | 0.048978 | E2F6,FZD4,FZD7,GJA1,GSK3B,HNF1A,  MAPK1,MSH2,PIK3R4,PRKAR2B,RBL2  ,RRAS2,WNT5A |
| PI3K/AKT Signaling | 0.002344 | 0.048978 | FOXO1,FOXO3,GSK3B,HSP90AA1,HSP90AB1,  IFNAR1,IL20RA,IL6ST,ITGA2,MAP3K5,MAPK1,  PPM1L,PPP2CB,PPP2R5E,RRAS2 |
| Mismatch Repair in Eukaryotes | 0.002344 | 0.048978 | MCM9,MSH2,RFC1,RPA1 |
| **Brown module (70 correlated methylated CpG sites mapped genes)** | | | |
| *Ingenuity Canonical Pathways* | P-value | Adjusted p-value | Molecules |
| Notch Signaling | 0.00011 | 0.018197 | DTX1,MAML2,NOTCH1 |
| Regulation of beta-cell development | 0.000148 | 0.018197 | HNF1A,MAML2,NOTCH1 |
| Role of Macrophages, Fibroblasts and Endothelial Cells in Rheumatoid Arthritis | 0.000162 | 0.018197 | HNF1A,LRP5,NLK,PDGFA,  PRKCZ,TLR5 |
| Epithelial Adherens Junction Signaling | 0.000603 | 0.043652 | ARHGEF17,HNF1A,NOTCH1,TNS1 |
| WNT/beta-catenin Signaling | 0.000871 | 0.043652 | HNF1A,LRP5,NLK,RARG |
| Signaling by NOTCH1 | 0.000912 | 0.043652 | DTX1,MAML2,NOTCH1 |
| VDR/RXR Activation | 0.000912 | 0.043652 | LRP5,PDGFA,PRKCZ |
| **Blue module (205 correlated methylated CpG sites mapped genes)** | | | |
| *Ingenuity Canonical Pathways* | P-value | Adjusted p-value | Molecules |
| G-Protein Coupled Receptor Signaling | 6.17E-05 | 0.033113 | CCR7,FZD5,GNB2,GPR160,GRK5,  GRK6,HRH1,MAP2K2,MEGF6,PLCB3,  RAC1,RGS10,RGS16,S1PR4 |
| Chronic Myeloid Leukemia Signaling | 0.000234 | 0.041687 | CTBP2,FZD5,HDAC4,KLF4,MAP2K2,  PLCB3,RAC1,RPTOR |
| Opioid Signaling Pathway | 0.000234 | 0.041687 | CACNA1H,GNB2,GRK5,GRK6,  MAP2K2,RAC1,RGS10,RGS16 |

**Supplementary Table S3:** The overlapping genes in asthma-severity associated co-methylated modules and co-expressed modules. The blue co-methylation module 327 CpG sites mapped to 205 genes (22 DEGs); the brown co-methylation module 95 CpG sites mapped to 70 genes (13 DEGs) and the turquoise co-methylation module 1382 CpG sites mapped to 723 genes (74 DEGs).

| **Co-methylated module** | | | | |
| --- | --- | --- | --- | --- |
| DMCs | | Blue (327) | Brown (95) | Turquoise (1382) |
| DEGs | | (22) | (70) | (74) |
| **Co-expression module** | **Turquoise** (785) | 2(ns) | 2 (ns) | **24(1.5e-17)** |
|  | **Brown** (140) | 2 (7.8e-03) | **5(**9.6e**-09)** | 5 (8.9e-05) |
|  | **Green** (103) | 1 (ns) | **2(**1.5e**-03)** | 3 (4.5e-03) |
|  | **Red (61)** | 1(ns) | 0 (ns) | **2 (0.017)** |
|  | Black (32) | 0 (ns) | 0 (ns) | 1 (ns) |
|  | **Blue** (598) | **14 (**1.5e**-17)** | 4 (2.7e-04) | **28(**1.4e-25**)** |
|  | **Yellow** (117) | 2 (ns) | 0 (ns) | **5 (3.8e-05)** |

**Supplementary Table S4**. List of significant pathways for genes that were correlated with the asthma-associated turquoise, brown and blue co-expression module eigenvectors. The correlated DEGs of each module with p-value < 0.01were included as input for pathway analysis.

| **Turquoise module (785 correlated differentially expressed genes)** | | | |
| --- | --- | --- | --- |
| *Ingenuity Canonical Pathways* | P-value | Adjusted p-value | Molecules |
| RHO GTPase cycle | 3.24E-09 | 2.95E-06 | ABCD3,ABI2,ACBD5,ARHGAP28,ARHGAP29,ARHGAP42,  ARHGAP5,ARHGEF12,ARHGEF26,ARL13B,BLTP3B,CCDC115,  CDC42BPA,CFTR,CPD,CPNE8,FAM169A,FILIP1,FRS2,GJA1,HSP90AA1,  HSP90AB1,KIDINS220,KTN1,LEMD3,MACO1,MPP7,MTR,MYO9A,  PAK2,PARD6B,PCDH7,PREX2,PTPN13,RNF20,RRAS2,SH3RF1,TMPO,  TOR1AIP1,VAPB,VAV3 |
| Generic Transcription Pathway | 1.2E-05 | 0.0036 | ESRRG,NR1D2,NR3C1,NR3C2,RBPJ,ZFP14,ZFP90,  ZIK1,ZKSCAN1,ZNF141,ZNF154,ZNF33B,ZNF382,ZNF418,  ZNF430,ZNF470,ZNF486,ZNF549,ZNF551,ZNF568,ZNF610,  ZNF619,ZNF627,ZNF660,ZNF667,ZNF671,ZNF677,ZNF678,  ZNF713,ZNF770,ZNF772,ZNF773 |
| Transcriptional Regulatory Network in Embryonic Stem Cells | 2.82E-05 | 0.0045 | BMPR1A,BMPR1B,BMPR2,FZD4,FZD7,GSK3B,  IL6ST,LIFR,MAPK1,MEIS1,PIK3R4,RRAS2,SET,  SMAD2,SMAD5,TGFBR3,WNT5A |
| Class I peroxisomal membrane protein import | 3.02E-05 | 0.0045 | ABCD2,ABCD3,ACBD5,PEX12,PEX19,PEX3 |
| Human Embryonic Stem Cell Pluripotency | 3.72E-05 | 0.0045 | BMP3,BMPR1A,BMPR1B,BMPR2,FOXO1,  FZD4,FZD7,GSK3B,HSP90AA1,HSP90AB1,ID4,LIFR,  MAPK1,PIK3R4,PRKCE,RRAS2,SMAD2,SMAD5,WNT5A |
| Mouse Embryonic Stem Cell Pluripotency | 3.98E-05 | 0.0045 | BMPR1A,BMPR2,FZD4,FZD7,GSK3B,HNF1A,  ID4,IL6ST,LIFR,MAPK1,PIK3R4,RRAS2,SMAD5 |
| Xenobiotic Metabolism Signaling | 4.07E-05 | 0.0045 | ALDH1A1,ALDH5A1,ALDH6A1,ALDH9A1,ARNT,  CAMK2D,ESD,FMO2,FMO3,GSTA1,GSTA2,GSTA3,  HS2ST1,HSP90AA1,HSP90AB1,MAP3K5,MAPK1,  NCOA1,PIK3R4,PPM1L,PPP2CB,PPP2R5E,PRKCE,RRAS2 |
| WNT/beta-catenin Signaling | 5.5E-05 | 0.005 | BMPR2,CDH12,CDH2,CSNK1G1,CSNK2A1,  CSNK2A2,FZD4,FZD7,GJA1,GSK3B,HNF1A,LRP6,PPM1L,  PPP2CB,PPP2R5E,TGFBR3,WNT5A |
| Mitotic Roles of Polo-Like Kinase | 5.75E-05 | 0.005 | ANAPC1,CDC16,HSP90AA1,HSP90AB1,PPM1L,  PPP2CB,PPP2R5E,RAD21,SMC3,WEE1 |
| Role of NANOG in Mammalian Embryonic Stem Cell Pluripotency | 6.17E-05 | 0.005 | BMP3,BMPR1A,BMPR1B,BMPR2,FZD4,FZD7,  GSK3B,IL6ST,LIFR,MAPK1,PIK3R4,RRAS2,SMAD5,WNT5A |
| Xenobiotic Metabolism CAR Signaling Pathway | 6.61E-05 | 0.005 | ALDH1A1,ALDH5A1,ALDH6A1,ALDH9A1,FMO2,  FMO3,GSTA1,GSTA2,GSTA3,HS2ST1,HSP90AA1,  HSP90AB1,MAPK1,NCOA1,PPM1L,PPP2CB,PPP2R5E,PRKCE |
| Xenobiotic Metabolism AHR Signaling Pathway | 0.0001 | 0.0079 | ALDH1A1,ALDH5A1,ALDH6A1,ALDH9A1,  ARNT,GSTA1,GSTA2,GSTA3,HSP90AA1,  HSP90AB1,NCOA1 |
| PTEN Signaling | 0.0001 | 0.0079 | BMPR1A,BMPR1B,BMPR2,CSNK2A1,CSNK2A2,  FOXO1,FOXO3,GSK3B,INSR,ITGA2,MAPK1,PREX2,  PTK2,RRAS2,TGFBR3 |
| Role of Osteoblasts, Osteoclasts and Chondrocytes in Rheumatoid Arthritis | 0.0002 | 0.0117 | BMP3,BMPR1A,BMPR1B,BMPR2,CSNK1G1,  FOXO1,FZD4,FZD7,GSK3B,HNF1A,IL33,LRP6,MAP3K5,  MAPK1,NFATC3,PIK3R4,SMAD5,TAB2,WNT5A |
| Aryl Hydrocarbon Receptor Signaling | 0.0002 | 0.0123 | ALDH1A1,ALDH5A1,ALDH6A1,ALDH9A1,  ARNT,GSTA1,GSTA2,GSTA3,HSP90AA1,HSP90AB1,  MAPK1,NCOA7,NFIA,NFIB,RBL2 |
| PXR/RXR Activation | 0.0002 | 0.0125 | ALDH1A1,FOXA2,FOXO1,GSTA1,GSTA2,  INSR,NCOA1,NR3C1,PRKAR2B |
| Regulation of the Epithelial-Mesenchymal Transition Pathway | 0.0002 | 0.0125 | APH1B,CDH12,CDH2,FRS2,FZD4,FZD7,  GSK3B,HNF1A,MAPK1,MET,PARD6B,PIK3R4,PYGO1,  RBPJ,RRAS2,SMAD2,WNT5A |
| NFE2L2 regulating anti-oxidant/detoxification enzymes | 0.0002 | 0.0134 | GCLM,GSTA1,GSTA2,GSTA3,TXNRD1 |
| Cellular response to heat stress | 0.0003 | 0.0138 | CAMK2D,EEF1A1,GSK3B,HSP90AA1,  HSP90AB1,HSPA4L,MAPK1,NUP133,NUP153,  RPA1,ST13 |
| Bile acid and bile salt metabolism | 0.0004 | 0.0181 | CH25H,CYP39A1,NCOA1,OSBPL6,  OSBPL9,SLC27A2 |
| STAT3 Pathway | 0.0004 | 0.0186 | BMPR1A,BMPR1B,BMPR2,IFNAR1,  IL20RA,IL6ST,INSR,MAP3K20,MAPK1,RRAS2,  SOCS5,SOCS6,TGFBR3 |
| HIPPO signaling | 0.0004 | 0.0186 | CRB1,PPM1L,PPP2CB,PPP2R5E,SAV1,SMAD2,SMAD5,TEAD1,TP53BP2,YAP1 |
| Iron homeostasis signaling pathway | 0.0005 | 0.0199 | ABCB10,ARNT,BMP3,BMPR1A,BMPR1B,BMPR2,CP,MAPK1,SLC11A2,  SLC40A1,SMAD2,SMAD5,TWSG1 |
| Chaperone Mediated Autophagy | 0.0005 | 0.0213 | ARL13B,CFTR,EEF1A1,HSP90AA1,  HSP90AB1 |
| IGF-1 Signaling | 0.0006 | 0.0229 | CSNK2A1,CSNK2A2,FOXO1,FOXO3,MAPK1,  PIK3R4,PRKAR2B,PTK2,RRAS2,SOCS5,SOCS6 |
| Role of WNT/GSK-3beta Signaling in the Pathogenesis of Influenza | 0.0009 | 0.0316 | CSNK1G1,FZD4,FZD7,GSK3B,HNF1A,  IFNAR1,NCOA1,NCOA4,WNT5A |
| Formaldehyde Oxidation II (Glutathione-dependent) | 0.0010 | 0.0331 | ADH5,ESD |
| Role of BRCA1 in DNA Damage Response | 0.001122 | 0.0331 | ARID2,BRD7,E2F6,FANCF,HLTF,MSH2,  RBL2,RFC1,RPA1 |
| Pulmonary Fibrosis Idiopathic Signaling Pathway | 0.0011 | 0.0331 | AEBP2,BMPR2,CDH2,EFNB2,FOXO1,FOXO3,  FZD4,FZD7,GSK3B,HNF1A,IL6ST,ITGA2,MAPK1,  PIK3R4,PTK2,RBPJ,RPS6KA5,RRAS2,SMAD2,TGFBR3,  WNT5A,YAP1 |
| Myelination Signaling Pathway | 0.0011 | 0.0331 | APH1B,ARHGAP5,ASCL1,BMP3,BMPR1A,  BMPR1B,BMPR2,FZD4,FZD7,GSK3B,ID4,ITGA2,  MAPK1,MBP,NFATC3,PIK3R4,PRKAR2B,PTK2,  RRAS2,SMAD2,SMAD5,WNT5A |
| Amyloid Processing | 0.001 | 0.0346 | APH1B,CSNK2A1,CSNK2A2,GSK3B,  MAPK1,PRKAR2B,PRKCE |
| Transport of bile salts and organic acids, metal ions and amine compounds | 0.0014 | 0.0407 | CP,SLC11A2,SLC13A2,SLC13A3,  SLC22A4,SLC39A10,SLC39A6,SLC40A1,SLC44A5 |
| Cardiomyocyte Differentiation via BMP Receptors | 0.0015 | 0.0416 | BMPR1A,BMPR1B,BMPR2,MEF2C,SMAD5 |
| Xenobiotic Metabolism PXR Signaling Pathway | 0.001 | 0.0467 | ALDH1A1,ALDH5A1,ALDH6A1,ALDH9A1  ,CAMK2D,ESD,GSTA1,GSTA2,GSTA3,HS2ST1,  HSP90AA1,HSP90AB1,NCOA1,PRKAR2B,PRKCE |
| FOXO-mediated transcription of cell cycle genes | 0.0018 | 0.0467 | FOXO1,FOXO3,RBL2,SMAD2 |
| Adipogenesis pathway | 0.001 | 0.0478 | BMPR1A,BMPR1B,BMPR2,FOXO1,FZD4,  FZD7,KAT6B,KAT7,RUNX1T1,SENP2,  SMAD5,WNT5A |
| Sertoli Cell-Germ Cell Junction Signaling Pathway (Enhanced) | 0.0020 | 0.0489 | CDH2,CLDN16,CLDN8,GJA1,GSK3B,  ITGA2,MAP3K5,MAPK1,PIK3R4,PPM1L,PPP2CB,  PPP2R5E,PRKAR2B,PTK2,RRAS2,SMAD2,SP3 |
| NRF2-mediated Oxidative Stress Response | 0.0021 | 0.0489 | ABCC4,CYP2U1,DNAJB4,DNAJC5B,  GCLM,GSK3B,GSTA1,GSTA2,GSTA3,HSP90AA1,HSP90AB1,  MAP3K5,MAPK1,PIK3R4,  PRKCE,RRAS2,TXNRD1 |
| EGR2 and SOX10-mediated initiation of Schwann cell myelination | 0.0021 | 0.0489 | ADGRV1,MBP,TEAD1,UTRN,YAP1 |
| Ovarian Cancer Signaling | 0.0022 | 0.0489 | E2F6,FZD4,FZD7,GJA1,GSK3B,HNF1A,MAPK1,MSH2,  PIK3R4,PRKAR2B,RBL2,RRAS2,WNT5A |
| PI3K/AKT Signaling | 0.0023 | 0.0489 | FOXO1,FOXO3,GSK3B,HSP90AA1,HSP90AB1,  IFNAR1,IL20RA,IL6ST,ITGA2,MAP3K5,MAPK1,  PPM1L,PPP2CB,PPP2R5E,RRAS2 |
| Mismatch Repair in Eukaryotes | 0.002344 | 0.0489 | MCM9, MSH2,RFC1,RPA1 |
| **Brown module (140 correlated differentially expressed genes)** | | | |
| *Ingenuity Canonical Pathways* | P-value | Adjusted p-value | Molecules |
| Axonal Guidance Signaling | 4.17E-05 | 0.020 | EFNB3,GLI3,GNG7,ITGB5,KEL,PLXNA2,PRKCZ,  RASD2,SEMA5A,SLIT2,WNT10A,WNT5B |
| **Yellow module (117 correlated differentially expressed genes)** | | | |
| *Ingenuity Canonical Pathways* | P-value | Adjusted p-value | Molecules |
| Colanic Acid Building Blocks Biosynthesis | 3.63E-05 | 0.0067 | GALE,GMDS,GMPPA |
| 1,25-dihydroxyvitamin D3 Biosynthesis | 6.76E-05 | 0.006 | CYP27B1,CYP2R1 |
| XBP1(S) activates chaperone genes | 8.13E-05 | 0.0067 | DNAJB11,KDELR3,SRPRB,XBP1 |
| Unfolded Protein Response (UPR) | 8.13E-05 | 0.0067 | CREB3L1,CREB3L4,XBP1 |
| O-linked glycosylation | 0.0001 | 0.0125 | B3GNT6,GALNT10,GALNT12,  GCNT3,MUC5AC |
| **Red module (61 correlated differentially expressed genes)** | | | |
| *Ingenuity Canonical Pathways* | P-value | Adjusted p-value | Molecules |
| Pathogen Induced Cytokine Storm Signaling Pathway | 3.63E-06 | 0.001175 | CXCR4,GZMB,IL18R1,IL18RAP,IL1R2,  MLKL,PRF1,SOCS3 |
| Activin Inhibin Signaling Pathway | 1.7E-05 | 0.0021 | CXCR4,FOSB,FOSL2,IL18RAP,IL1R2,MXD1 |
| STAT3 Pathway | 2.14E-05 | 0.0021 | IL18R1,IL18RAP,IL1R2,IL7R,SOCS3 |
| Role of Chondrocytes in Rheumatoid Arthritis Signaling Pathway | 2.63E-05 | 0.0021 | CXCR4,IL18R1,IL18RAP,IL1R2,MLKL |
| Tetrahydrofolate Salvage from 5,10-methenyltetrahydrofolate | 6.03E-05 | 0.0038 | MTHFD1L,MTHFD2 |
| Immunogenic Cell Death Signaling Pathway | 7.41E-05 | 0.0039 | GZMA,GZMB,MLKL,PRF1 |
| Histidine Degradation III | 0.0001 | 0.007413 | MTHFD1L,MTHFD2 |
| IL-13 Signaling Pathway | 0.0002 | 0.0074 | DUSP1,FOSL2,HBEGF,SOCS3 |
| Folate Transformations I | 0.0002 | 0.0074 | MTHFD1L,MTHFD2 |
| p38 MAPK Signaling | 0.0002 | 0.0074 | DUSP1,IL18RAP,IL1R2,PLA2G4A |
| IL-10 Signaling | 0.0005 | 0.0169 | DUSP1,IL18RAP,IL1R2,SOCS3 |
| Granzyme B Signaling | 0.0007 | 0.0190 | GZMB,PRF1 |
| Glioma Invasiveness Signaling | 0.0008 | 0.02138 | CD44,PLAUR,RHOH |
| Th1 and Th2 Activation Pathway | 0.0009 | 0.02138 | CXCR4,IL18R1,NFIL3,SOCS3 |
| Macrophage Alternative Activation Signaling Pathway | 0.0012 | 0.0275 | DUSP1,IL1R2,SOCS3,THBS1 |
| PI3K/AKT Signaling | 0.0015 | 0.0309 | IL18R1,IL18RAP,IL1R2,IL7R |
| Tumoricidal Function of Hepatic Natural Killer Cells | 0.0016 | 0.0309 | GZMB,PRF1 |
| Cardiac Hypertrophy Signaling (Enhanced) | 0.0022 | 0.0407 | IL18R1,IL18RAP,IL1R2,IL7R,  PDE3B,PDE4B |
| Cargo recognition for clathrin-mediated endocytosis | 0.0023 | 0.0407 | AREG,HBEGF,IL7R |
| cAMP-mediated signaling | 0.0028 | 0.0426 | DUSP1,PDE3B,PDE4B,RGS2 |
| IL-12 Signaling and Production in Macrophages | 0.0028 | 0.0426 | ETS2,NFIL3,SOCS3,THBS1 |
| Sertoli Cell-Germ Cell Junction Signaling Pathway (Enhanced) | 0.0028 | 0.0426 | FOSB,FOSL2,IL18RAP,IL1R2 |
| Glucocorticoid Receptor Signaling | 0.0032 | 0.0446 | DUSP1,IL18R1,IL18RAP,IL1R2,IL7R,  PLA2G4A |
| Coagulation System | 0.0033 | 0.0457 | PLAUR,THBD |
| Th1 Pathway | 0.0036 | 0.0467 | IL18R1,NFIL3,SOCS3 |
| Interleukin-1 family signaling | 0.0039 | 0.0467 | IL18R1,IL18RAP,IL1R2 |
| RAF/MAP kinase cascade | 0.0040 | 0.0467 | AREG,DUSP1,HBEGF,RASGRP4 |
| Clathrin-mediated endocytosis | 0.0041 | 0.0467 | AREG,HBEGF,IL7R |
| IL-6 Signaling | 0.0042 | 0.0467 | IL18RAP,IL1R2,SOCS3 |
| RAR Activation | 0.0042 | 0.0467 | DUSP1,PDE3B,PDE4B,RHOH,SOCS3 |
| **Green module (103 correlated differentially expressed genes)** | | | |
| *Ingenuity Canonical Pathways* | P-value | Adjusted p-value | Molecules |
| Protein Kinase A Signaling | 6.03E-06 | 0.0031 | DUSP16,DUSP4,FLNB,HHAT,ITPR1,MYH10,  NFATC2,PLCH1,PTPRT,SMAD3 |
| Pre-NOTCH Expression and Processing | 2.45E-05 | 0.006 | ATP2A2,MAML2,MAML3,NAD ,NOTCH2 |
| Signaling by NOTCH2 | 0.0003 | 0.0398 | MAML2,MAML3,NOTCH2 |
| Signaling by NOTCH4 | 0.0003 | 0.0398 | MAML2,MAML3,NOTCH2,SMAD3 |
| Notch Signaling | 0.0004 | 0.0398 | MAML2,MAML3,NOTCH2 |
| C-type lectin receptors (CLRs) | 0.0004 | 0.0398 | ITPR1,MUC5B,NAD ,NFATC2,PDPK1 |
| Beta-Alanine Degradation I | 0.0006 | 0.0398 | ABAT,NAD |
| 4-aminobutyrate Degradation I | 0.0006 | 0.0398 | ABAT,NAD |
| Transcriptional regulation by RUNX3 | 0.0006 | 0.0398 | MAML2,MAML3,SMAD3,ZFHX3 |
| Chronic Myeloid Leukemia Signaling | 0.0008 | 0.0436 | CDKN1B,NFATC2,NOTCH2,PLCH1,  SIN3A,SMAD3 |
| Glutamate Degradation III (via 4-aminobutyrate) | 0.0009 | 0.0457 | ABAT,NAD |

**Supplementary Table S5**: Identification of key differentially methylated CpGs in asthmatic subjects using Boruta machine method.

| CpG site | Gene Symbol | Meanimp | Medianimp | Minimp | Maximp | Normhits | Decision |
| --- | --- | --- | --- | --- | --- | --- | --- |
| cg00406211 | GRK5 | 8.258791 | 8.413396 | 5.038321 | 10.16921 | 1 | Confirmed |
| cg00579868 | WIPI2 | 8.038384 | 8.087493 | 5.545672 | 9.76241 | 1 | Confirmed |
| cg05194102 | SLC9A3R2 | 6.768102 | 6.918323 | 3.962289 | 8.992155 | 1 | Confirmed |
| cg07418114 | NTRK1 | 5.640159 | 5.664126 | 3.936186 | 8.366417 | 1 | Confirmed |
| cg14015211 | TLR5 | 5.620547 | 5.710587 | 2.927153 | 7.647497 | 1 | Confirmed |
| cg23752651 | TNFRSF1A | 5.197159 | 5.209783 | 3.064851 | 6.962165 | 0.969697 | Confirmed |
| cg21516291 | SLC35C2 | 5.114512 | 5.154037 | 3.033912 | 6.790452 | 0.959596 | Confirmed |
| cg12978575 | ZNF274 | 4.702164 | 4.726089 | 2.652102 | 6.550079 | 0.919192 | Confirmed |
| cg10893014 | TEAD3 | 4.336096 | 4.452397 | 2.122116 | 6.581303 | 0.909091 | Confirmed |
| cg17119907 | NOS1 | 4.128879 | 4.120946 | 1.627959 | 6.180835 | 0.868687 | Confirmed |
| cg10914558 | NOS1 | 3.97904 | 4.061839 | 1.769189 | 5.994385 | 0.878788 | Confirmed |
| cg18416096 | SYNPO | 3.920356 | 3.917429 | 1.866399 | 5.64174 | 0.828283 | Confirmed |
| cg00004883 | LDHD | 3.895874 | 3.97992 | 1.497922 | 5.654077 | 0.808081 | Confirmed |
| cg03130962 | CCHCR1 | 3.678545 | 3.792223 | 1.475744 | 5.516299 | 0.767677 | Confirmed |
| cg14375985 | CTBP2 | 3.607161 | 3.6857 | 1.276771 | 5.319597 | 0.767677 | Confirmed |
| cg21949830 | SLC43A2 | 3.500756 | 3.596603 | 0.888644 | 5.069066 | 0.707071 | Confirmed |
| cg10528482 | SLC9A3 | 3.279646 | 3.196028 | 1.32348 | 5.570482 | 0.616162 | Confirmed |
| cg24054525 | TRPM4 | 2.660314 | 2.792764 | 0.490618 | 4.563572 | 0.505051 | Confirmed |

**Supplementary Table S6:** Identification of key differentially expressed genes in asthmatic subjects using Boruta machine method.

| **Gene Symbol** | **Meanimp** | **Medianimp** | **Minimp** | **Maximp** | **Normhits** | **Decision** |
| --- | --- | --- | --- | --- | --- | --- |
| KCNA1 | 4.780625 | 4.886512 | 3.279242 | 6.229612 | 0.989899 | Confirmed |
| ALOX15B | 5.549791 | 5.674521 | 3.256737 | 7.270339 | 0.989899 | Confirmed |
| SLC9B2 | 6.25425 | 6.219365 | 3.39142 | 8.398586 | 0.989899 | Confirmed |
| LCA1 | 4.321674 | 4.255555 | 2.215217 | 6.056369 | 0.969697 | Confirmed |
| DNAJC1 | 4.635114 | 4.628444 | 3.40354 | 6.126086 | 0.959596 | Confirmed |
| TMEM45A | 4.416359 | 4.411991 | 2.614744 | 6.253336 | 0.959596 | Confirmed |
| CHGA | 4.47365 | 4.57107 | 1.223417 | 6.120226 | 0.959596 | Confirmed |
| ZFP36 | 4.009432 | 4.017632 | 2.460844 | 5.796787 | 0.939394 | Confirmed |
| OR51E1 | 4.239531 | 4.254749 | 2.218721 | 5.905282 | 0.939394 | Confirmed |
| CHST10 | 4.205948 | 4.281096 | 1.675528 | 6.14988 | 0.929293 | Confirmed |
| BDKRB1 | 4.146553 | 4.247571 | 1.451664 | 5.649851 | 0.919192 | Confirmed |
| FGFBP1 | 3.996063 | 4.016876 | 1.926633 | 5.796827 | 0.89899 | Confirmed |
| KLF4 | 3.801533 | 3.806285 | 1.851687 | 5.301746 | 0.878788 | Confirmed |
| CXCR4 | 3.630141 | 3.625021 | 0.792573 | 5.543582 | 0.848485 | Confirmed |
| SLC24A3 | 3.460631 | 3.599102 | 0.894901 | 4.835463 | 0.848485 | Confirmed |
| KCNJ2 | 3.855902 | 3.869968 | 1.591702 | 5.749643 | 0.848485 | Confirmed |
| NOB1 | 3.652297 | 3.673158 | 1.54229 | 5.320592 | 0.838384 | Confirmed |
| SCG5 | 3.535468 | 3.559889 | 1.67081 | 5.53159 | 0.828283 | Confirmed |
| RCAN3 | 3.575648 | 3.691197 | 1.054784 | 5.840029 | 0.818182 | Confirmed |
| SYT8 | 3.621134 | 3.729265 | 1.72005 | 5.125688 | 0.808081 | Confirmed |
| FAM174B | 3.385561 | 3.342915 | 1.529699 | 5.197734 | 0.79798 | Confirmed |
| ATXN1 | 3.447439 | 3.479776 | 0.638638 | 5.095094 | 0.787879 | Confirmed |
| LRIG1 | 3.294621 | 3.307754 | 1.441736 | 5.309201 | 0.787879 | Confirmed |
| PHACTR3 | 3.322571 | 3.264845 | 0.506639 | 4.871498 | 0.787879 | Confirmed |
| CEACAM5 | 3.437941 | 3.505715 | 1.02829 | 4.967089 | 0.777778 | Confirmed |
| INSR | 3.16862 | 3.127446 | 1.139103 | 4.866269 | 0.727273 | Confirmed |
| KCNQ3 | 3.085459 | 3.05444 | 0.712462 | 4.85781 | 0.707071 | Confirmed |
| FA2H | 3.048208 | 3.044412 | 0.830906 | 4.747847 | 0.69697 | Confirmed |

**Supplementary Table S7**: Pairwise correlation between DNA methylation of CpGs and expression annotated DEGs in BECs of asthmatic and control subjects in the discovery dataset.

| CPG | Gene | correlation | P-value |
| --- | --- | --- | --- |
| cg00406211 | GRK5 | -0.223 | 0.0478 |
| cg01975495 | SERPINE1 | 0.250 | 0.006 |
| cg02766259 | AACS | -0.221 | 0.016 |
| cg03441945 | ABAT | -0.225 | 0.014 |
| cg06128142 | GPT2 | 0.508 | 2.45E-06 |
| cg08801887 | TCIRG1 | 0.224 | 0.047 |
| cg09048665 | WDR90 | -0.22 | 0.016 |
| cg10002185 | TRPM4 | 0.317 | 0.0045 |
| cg10290200 | FLNC | -0.46 | 0.00002 |
| cg10336131 | CNIH2 | 0.24 | 0.0314 |
| cg10528482 | SLC9A3 | 0.48 | 4.0E-08 |
| cg10672136 | TPO | 0.28 | 0.001 |
| cg11419403 | CLPTM1L | -0.25 | 0.006 |
| cg11703729 | FRMD4A | -0.35 | 0.001 |
| cg13345353 | GAL3ST2 | -0.42 | 0.0001 |
| cg13997788 | EPAS1 | -0.38 | 0.0005 |
| cg14015211 | TLR5 | -0.51 | 2.5E-09 |
| cg14185918 | KLF4 | -0.29 | 0.001 |
| cg15012607 | ETHE1 | 0.3 | 0.0075 |
| cg15562220 | SCGN | -0.3 | 0.0009 |
| cg16393012 | ARHGDIB | -0.29 | 0.008 |
| cg17012160 | FMN2 | -0.29 | 0.01 |
| cg17602126 | HEYL | -0.35 | 0.00009 |
| cg17945560 | TINAGL1 | 0.29 | 0.0016 |
| cg18181229 | PBX1 | -0.27 | 0.01482 |
| cg19212949 | PEG3 | 0.40 | 8.71E-06 |
| cg19651003 | BSG | -0.25 | 0.0254 |
| cg20456258 | DGKQ | 0.224 | 0.047 |
| cg20491914 | KCNK3 | -0.29 | 0.007 |
| cg21385480 | LRIG1 | -0.5 | 8.8E-09 |
| cg23556108 | BCL11A | -0.24 | 0.03376 |
| cg23817893 | CCDC81 | -0.33 | 0.002 |
| cg25267808 | MAML2 | -0.22 | 0.04468 |
| cg25477769 | HNF1A | -0.42 | 1.8E-06 |
| cg26639146 | CD9 | -0.29 | 0.0016 |

**Supplementary Table S8:** Results of mediation analysis for asthma severity

| **CpG** | **Gene** | **Total effect** | | **Direct effect (DE)** | | **Mediational effect (ME)** | | |
| --- | --- | --- | --- | --- | --- | --- | --- | --- |
|  |  | $\boldsymbol{\beta}$ **(95% CI)** | **p-value** | $\boldsymbol{\beta}$ **(95% CI)** | **p-value** | $\boldsymbol{\beta}$ **(95% CI)** | **p-value** | **Mediation proportion** |
| cg00406211 | GRK5 | -0.41(-0.512, -0.3) | 2E-16 | -0.38(-0.49,-0.27) | 2E-16 | -0.028(-0.086,01) | 0.11 | 6.78 |
| cg02766259 | AACS | -0.69(-1.05, -0.34) | 2E-16 | -0.51(-0.90,-0.16) | 0.004 | -0.19(-0.359,-0.04 ) | **0.006** | 26.82 |
| cg03441945 | ABAT | 0.77(0.33,1.19) | 0.002 | 0.65(0.18,1.1) | 0.004 | 0.12(0.01,0.28 ) | **0.022** | 15.4 |
| cg06128142 | GPT2 | 0.528(0.326,0.7) | 2E-16 | 0.369(0.1202,0.6) | 2E-16 | 0.159(0.033,0.291) | **0.012** | 30.2 |
| cg08801887 | TCIRG1 | 0.650(0.398,0.89) | 2E-16 | 0.509(0.250,0.79) | 0.002 | 0.142(0.024,0.271) | **0.02** | 21.76 |
| cg09048665 | WDR90 | -0.48(-0.8,-0.18) | 2E-16 | -0.35(-0.65,-0.04) | 0.028 | -0.14( -0.29, -0.03) | **0.002** | 28.17 |
| cg10002185 | TRPM4 | 0.33(0.179,0.47) | 2E-16 | 0.259(0.0857,0.4) | 0.002 | 0.071(0.007,0.151) | **0.026** | 21.38 |
| cg10336131 | CNIH2 | 0.472(0.225,0.72) | 0.002 | 0.4(0.117,0.66) | 0.004 | 0.074(0.005,0.211) | **0.03** | 15.66 |
| cg10672136 | TPO | 1.39(0.86,1.85) | 2E-16 | 1.11(0.59,1.60) | 2E-16 | 0.27(0.09,0.50) | **2E-16** | 19.8 |
| cg11419403 | CLPTM1L | -0.5(-0.84,-0.16) | 2E-16 | -0.35(-0.72,-0.03) | 0.042 | -0.14(-0.279,-0.02) | **0.018** | 28.77 |
| cg11703729 | FRMD4A | 0.3175(0.213,0.42 | 2E-16 | 0.259(0.14,0.37) | 2E-16 | 0.058(-.002,0.131) | 0.056 | 18.37 |
| cg13345353 | GAL3ST2 | 0.610(0.353,0.87) | 2E-16 | 0.529(0.228,0.83) | 2E-16 | 0.08(-0.029,0.241) | 0.15 | 13.35 |
| cg13997788 | EPAS1 | 0.374(0.260,0.49) | 2E-16 | 0.294(0.161,0.42) | 2E-16 | 0.08(0.026,0.151) | **0.008** | 21.47 |
| cg14185918 | KLF4 | -0.40(-0.70,-0.11) | 0.01 | -0.22(-0.49,0.08) | 0.13 | -0.19(-0.3, -0.09) | **2E-16** | 0.459 |
| cg15012607 | ETHE1 | 0.468(0.109,0.85) | 0.024 | 0.32(-0.052,0.74) | 0.08 | 0.15(0.0236,0.291) | **0.008** | 31.67 |
| cg15562220 | SCGN | 0.54(0.15,0.87) | 0.01 | 0.27(-0.09,0.63) | 0.15 | 0.27(0.13,0.42) | **2E-16** | 49.89 |
| cg16393012 | ARHGDIB | -0.26(-0.39,-0.12) | 2E-16 | -0.20(-0.34,-0.06) | 0.01 | -0.06(-0.112,-0.01) | **0.008** | 22.22 |
| cg17012160 | FMN2 | 0.366(0.13,0.58) | 0.002 | 0.290(0.065,0.51) | 0.006 | 0.076(0.021,0.141) | **0.01** | 20.82 |
| cg17602126 | HEYL | 1.09(0.61,1.52 ) | 2E-16 | 0.71(0.22,1.14) | 2E-16 | 0.31(0.19,0.63) | **2E-16** | 35.0 |
| cg17945560 | TINAGL1 | 0.34(0.06,0.59) | 0.016 | 0.076(-0.15,0.28) | 0.52 | 0.26(0.093,0.44) | **2E-16** | 77.5 |
| cg18181229 | PBX1 | 0.357(0.194,0.51) | 2E-16 | 0.247(0.07,0.41) | 0.008 | 0.110(0.049,0.21) | **2E-16** | 30.86 |
| cg19212949 | PEG3 | -0.22(-0.353,-0.06) | 0.004 | -0.14(-0.278,0.01) | 0.072 | -0.08(-0.14,-0.031) | **2E-16** | 37.95 |
| cg19651003 | BSG | -0.36(-0.58,-0.14) | 2E-16 | -0.3(-0.533,-0.07) | 0.008 | -0.07(-0.1378,01) | **0.048** | 18.11 |
| cg20456258 | DGKQ | 0.22(0.105,0.31) | 2E-16 | 0.168(0.05,0.28) | 0.002 | 0.047(0.007,0.091) | **0.018** | 21.99 |
| cg20491914 | KCNK3 | 0.55(0.197,0.88) | 0.004 | 0.288(-0.09,0.63) | 0.136 | 0.26(0.10,0.45) | **0.002** | 47.91 |
| cg23556108 | BCL11A | 0.441(0.26,0.63) | 2E-16 | 0.331(0.123,0.52) | 0.006 | 0.11(0.032,0.211) | **0.006** | 24.84 |
| cg23817893 | CCDC81 | 0.504(0.268,0.72) | 2E-16 | 0.369(0.114,0.62) | 2E-16 | 0.14(0.04,0.241) | **0.012** | 26.84 |
| cg25267808 | MAML2 | 0.211(0.12,0.3) | 2E-16 | 0.179(0.078,0.27) | 2E-16 | 0.031(0.006,0.071) | **0.006** | 14.85 |

We performed mediation analysis to assess the parts of the relationship between methylation of CpG and asthma severity level is mediated via differential expression of genes. ME denotes causal mediational effect (indirect effect of each CpG on asthma-severity that goes through mediating gene). The values in bold show genes that are significantly mediating the DNA methylation effect and asthma severity association. P-values show the significance of total effect (asthma severity $\to$ methylation), mediational effect (CpG methylation →gene expression → asthma severity) and direct effect (CpG methylation $\to$ asthma severity) adjusting sex, age and ancestry. The values in bold show genes that are significantly mediating the DNA methylation effect and asthma severity association.

**Supplementary Table S9**: Results of mediation analysis for lung function (FEV1)

| **CpG** | **Gene** | $\boldsymbol{\beta}$ **(95% CI)** | **p-value** | $\boldsymbol{\beta}$ **(95% CI)** | **P-value** | $\boldsymbol{\beta}$**(95% CI)** | **P-value** | **Mediation**  **proportion** |
| --- | --- | --- | --- | --- | --- | --- | --- | --- |
| cg00406211 | GRK5 | 0.34(0.15-0.5) | 2.00E-16 | 0.27(0.08,0.43) | 0.008 | 0.07(0.02,0.14) | 2.00E-16 | 19.24 |
| cg02766259 | AACS | 0.47(0.14, 0.78) | 0.004 | 0.33(0.01,0.67) | 0.048 | 0.13(0.03, 0.27) | 0.006 | 0.2866 |
| cg03441945 | ABAT | -0.45(-0.8,-0.10) | 0.01 | -0.41(-0.78, -0.04) | 0.028 | -0.04(-0.17, 0.04) | 0.306 | 0.0947 |
| cg06128142 | GPT2 | -0.61(-0.91,-0.32) | 2E-16 | -0.44(-0.76, -0.12) | 0.006 | -0.17(-0.34, 0) | 0.054 | 27.137 |
| cg08801887 | TCIRG1 | -0.63(-1.06,-0.22) | 0.002 | -0.52(-0.99, -0.05) | 0.032 | -0.12(-0.34, 0.06) | 0.182 | 18.35 |
| cg09048665 | WDR90 | 0.39( 0.09, 0.69) | 0.002 | 0.29(-0.01, 0.62) | 0.07 | 0.1(0.01, 0.22) | 0.03 | 0.2477 |
| cg10002185 | TRPM4 | -0.40(-0.6,-0.22) | 2E-16 | -0.25(-0.45, -0.06) | 0.008 | -0.15(-0.28, -0.05) | 0.006 | 37.8 |
| cg10336131 | CNIH2 | -0.56(-0.9,-0.24) | 0.002 | -0.41(-0.76, -0.08) | 0.012 | -0.16(-0.33, -0.02) | 0.016 | 27.73 |
| cg10672136 | TPO | -0.71(-1.19, -0.20) | 0.006 | -0.60(-1.1, -0.03) | 0.042 | -0.12(-0.33, 0.03) | 0.138 | 0.1652 |
| cg11419403 | CLPTM1L | 0.33(0.08, 0.59) | 0.014 | 0.18(-0.08,0.45) | 0.21 | 0.18(-0.08,0.45) | 0.21 | 0.4365 |
| cg11703729 | FRMD4A | -0.26(-0.42,-0.08) | 0.01 | -0.19(-0.37, -0.02) | 0.038 | -0.06(-0.15, 0.03) | 0.194 | 23.97 |
| cg13345353 | GAL3ST2 | -0.56(-0.96,-0.15) | 0.006 | -0.39(-0.80, 0.03) | 0.064 | -0.16(-0.40, 0.01) | 0.074 | 29.43 |
| cg13997788 | EPAS1 | -0.35(-0.51,-0.17) | 2E-16 | -0.27(-0.44, -0.09) | 0.012 | -0.07(-0.17, 0.01) | 0.098 | 21.1 |
| cg14185918 | KLF4 | 0.27(0.04,0.50) | 0.024 | 0.18(-0.05,0.39) | 0.116 | 0.09(0.026, 0.19) | 0.006 | 0.345 |
| cg15012607 | ETHE1 | -0.73(-1.19,-0.22) | 0.006 | -0.53(-1.01, -0.04) | 0.038 | -0.2(-0.38, -0.04) | 2E-16 | 26.97 |
| cg15562220 | SCGN | -0.30(-0.59,-0.02) | 0.034 | -0.13(-0.44,0.17 ) | 0.432 | -0.16(-0.29, -0.07) | 2E-16 | 0.549 |
| cg16393012 | ARHGDIB | 0.26(0.07-0.43) | 0.004 | 0.28(0.073,0.46) | 0.004 | -0.03(-0.09,0.04) | 0.502 | 9.65 |
| cg17012160 | FMN2 | -0.34(-0.62,-0.06) | 0.012 | -0.25(-0.53, 0.04) | 0.094 | -0.1(-0.21, -0.02) | 0.012 | 28.26 |
| cg17602126 | HEYL | -0.87(-1.26,-0.47) | 2E-16 | -0.67(-1.08,-0.28 ) | 0.002 | -0.2(-0.4,-0.03) | 0.018 | 0.225 |
| cg17945560 | TINAGL1 | -0.27(-0.53, 0.01) | 0.06 | -0.11(-0.36, 0.13) |  | -0.17(-0.27,-0.06) | 2E-16 | 0.612 |
| cg18181229 | PBX1 | -0.37(-0.62,-0.12) | 0.008 | -0.32(-0.58, -0.03) | 0.034 | -0.05(-0.18, 0.04) | 0.294 | 14.28 |
| cg19212949 | PEG3 | 0.09(-0.13-0.3) | 0.474 | -0.04(-0.27,0.2) | 0.77 | 0.13(0.02,0.21) | 0.018 | 149.19 |
| cg19651003 | BSG | 0.38(0.10-0.66) | 0.004 | 0.26(-0.03,0.55) | 0.084 | 0.12(0.02,0.25) | 0.02 | 32.49 |
| cg20456258 | DGKQ | -0.15(-0.29,-0.01) | 0.044 | -0.07(-0.21, 0.08) | 0.374 | -0.08(-0.16, -0.02) | 0.002 | 53.29 |
| cg20491914 | KCNK3 | -0.267(-0.68-0.2) | 0.238 | -0.01(-0.51,0.57) | 0.966 | -0.26(-0.58,-0.04) | 0.014 | 95.85 |
| cg23556108 | BCL11A | -0.51(-0.81,-0.21) | 0.006 | -0.50(-0.79, -0.2) | 0.002 | -0.004(-0.11, 0.11) | 0.916 | 0.727 |
| cg23817893 | CCDC81 | -0.57(-0.88,-0.24) | 2E-16 | -0.34(-0.70, 0.06) | 0.08 | -0.23(-0.44, -0.06) | 0.012 | 40.96 |

We performed mediation analysis to assess the parts of the relationship between methylation of CpG and asthma severity level is mediated via differential expression of genes. ME denotes causal mediational effect (indirect effect of each CpG on asthma-severity that goes through mediating gene). The values in bold show genes that are significantly mediating the DNA methylation effect and asthma severity association. P-values show the significance of total effect (FEV1 → methylation), mediational effect (CpG methylation →gene expression → FEV1) and direct effect (CpG methylation → FEV1) adjusting sex, age and ancestry. The values in bold show genes that are significantly mediating the DNA methylation effect and FEV1 association.

**Supplementary Table S10**: Datasets used in our study.

| **GEO ID** | **Experiment type** | **Data type** | **Sample size (asthmatics /controls)** | **Tissue type** | **Category** |
| --- | --- | --- | --- | --- | --- |
| GSE201872 | Illumina HumanMethylation450 BeadChip and Infinium MethylationEPIC | DNA methylation | 96/46 | BECs | Discovery set |
| GSE201955 | Expression by RNA sequencing | RNA-seq | 79/39 | BECs | Discovery set |
|  |  |  |  |  |  |
| GSE85568 | Illumina HumanMethylation450 BeadChip and Illumina HiSeq 2000 | DNA methylation | 74/41 | AECs | Validation set |
| GSE85567 | Expression by RNA sequencing | RNA-seq | 57/28 | ACEs | Validation set |

Note: there are 118 samples that have both methylation and gene expression profiles in BECs, and 81 samples have both methylation and gene expression profiles in AECs.
